# A randomised trial of the pharmacodynamic and pharmacokinetic effects of ticagrelor compared with clopidogrel in Hispanic patients with stable coronary artery disease

**DOI:** 10.1007/s11239-014-1135-9

**Published:** 2014-10-11

**Authors:** Matthew J. Price, Leonardo Clavijo, Dominick J. Angiolillo, Glenn Carlson, Richard Caplan, Renli Teng, Juan Maya

**Affiliations:** 1 10666 North Torrey Pines Road, Maildrop S1056, La Jolla, CA 92037 USA; 2 Division of Cardiology, Keck School of Medicine, University of Southern California, 1510 San Pablo Street, Suite 322, Los Angeles, CA 90033 USA; 3 Division of Cardiology, Department of Medicine, University of Florida College of Medicine-Jacksonville, Shands Jacksonville, 655 West 8th St, Jacksonville, FL 32209 USA; 4 AstraZeneca LP, 1800 Concord Pike, P.O. Box 15437, Wilmington, DE 19850-5437 USA

**Keywords:** Platelet reactivity, Ticagrelor, Clopidogrel, Hispanic, Coronary artery disease

## Abstract

The objective was to compare the pharmacodynamic (PD) and pharmacokinetic (PK) effects of ticagrelor with clopidogrel among subjects of Hispanic ethnicity, as the PD and PK effects of antiplatelet agents among Hispanics are not specifically known. This was a randomised, open-label, crossover PD/PK study of 40 Hispanic subjects with stable coronary artery disease (CAD). Subjects were allocated to either ticagrelor 180 mg loading dose (LD)/90 mg twice-daily maintenance dose (MD) followed by clopidogrel 600 mg LD/75 mg once-daily MD with an intervening washout period, or vice versa. The primary endpoint was on-treatment reactivity (OTR) at 2 h post-LD according to the VerifyNow P2Y12 test. OTR was significantly lower at 2 h post-LD with ticagrelor compared with clopidogrel (34 PRU vs. 201 PRU, least square means difference = −167 PRU [95 % CI, −197, −137], *P* < 0.001). OTR was also lower with ticagrelor at 30 min and 8 h post-LD (*P* < 0.001). The greater magnitude of antiplatelet effect with ticagrelor persisted after 7 days of MD (52 PRU [95 % CI, 30, 73] vs. 182 PRU [95 % CI, 160, 205], *P* < 0.001). Mean plasma concentration of ticagrelor and its active metabolite were greatest at 2 h post-LD, with similar levels at 2 h post-MD after 7 days of MD. Among Hispanic subjects with stable CAD, ticagrelor provides a more rapid onset of platelet inhibition and a significantly greater antiplatelet effect compared with clopidogrel during both the loading and maintenance phases of treatment.

**Electronic supplementary material:**

The online version of this article (doi:10.1007/s11239-014-1135-9) contains supplementary material, which is available to authorized users.

## Introduction

Coronary artery disease (CAD) and acute coronary syndrome (ACS) are leading causes of mortality in the United States [1]. Interventions that may reduce the incidence of ACS or mitigate its sequelae are therefore of substantial societal benefit. However, the populations evaluated in most randomised cardiovascular outcomes trials do not reflect the broad range of ethnicities to which the therapies studied may be subsequently applied in clinical practice. Engaging under-represented communities in research and identifying patient subgroups whose response to a given therapy may differ from that of the average patient in a trial are priorities for scientific research and investment [2]. Hispanics in the United States have higher rates of diabetes mellitus, metabolic syndrome and obesity than non-Hispanic whites, have a substantial burden of cardiovascular disease, and are a growing fraction of the national population. Ticagrelor is an oral platelet P2Y_12_-receptor antagonist that significantly reduces major cardiovascular events after ACS compared with clopidogrel, driven by reductions in myocardial infarction and cardiovascular death, without a significant increase in all-cause major bleeding, although non-coronary artery bypass grafting related bleeding is increased [3]. We sought to evaluate the antiplatelet effects of ticagrelor compared with clopidogrel in Hispanic subjects with established CAD.

## Materials and methods

### Study design

This was a randomised, open-label, crossover study conducted at six sites within the United States (See Online Appendix for study sites). The study was approved by the Institutional Review Boards at all sites and was conducted in accord with the provisions of the Declaration of Helsinki. All subjects provided written, informed consent (clinicaltrials.gov identifier, NCT01523366).

### Study population

Subjects were eligible to be enrolled if they were a Hispanic male or female ≥18 years of age, were receiving aspirin 75–100 mg daily maintenance dose (MD), and had documented stable CAD according to one of the following criteria: (a) current or history of stable angina with objective evidence of CAD; (b) prior myocardial infarction; or (c) prior surgical or percutaneous coronary revascularisation. Hispanic ethnicity was based upon self-identification. Major exclusion criteria included any indication for oral anticoagulation or dual antiplatelet therapy, and concomitant therapy with strong CYP3A4 inhibitors or inducers. Detailed inclusion and exclusion criteria and participating sites are listed in the Online Appendix.

### Study procedures

**Fig. 1:**
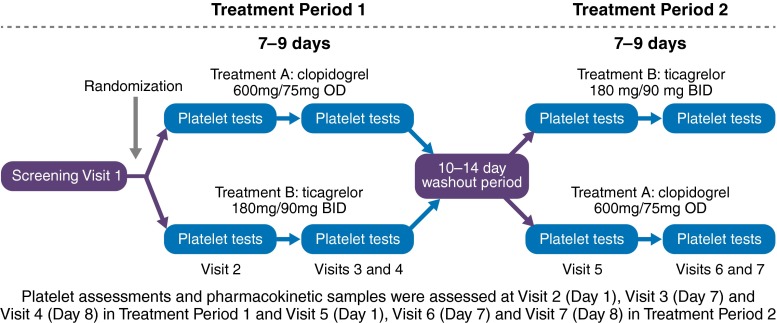
Study design. Hispanic subjects with CAD and treated with aspirin therapy were randomly assigned in a 1:1 fashion to one of two treatment sequences: either ticagrelor 180 mg LD and 90 mg twice-daily MD for 7–9 days, followed by a washout period and clopidogrel 600 mg LD and 75 mg once-daily MD for 7–9 days, or clopidogrel 600 mg LD and 75 mg once-daily MD for 7–9 days, followed by a washout period and ticagrelor 180 mg LD and 90 mg twice-daily MD for 7–9 days. Platelet reactivity assessment and ticagrelor plasma concentrations were measured at several timepoints around the LD and at the end of the MD phase. *CAD* coronary artery disease, *LD* loading dose, *MD* maintenance dose

Subjects were randomised 1:1 to receive one of two possible treatment sequences: either open-label clopidogrel in the first period followed by open-label ticagrelor in the second period or vice versa (Fig. 1). There was a 10–14 days washout between periods. Clopidogrel was administered as a 600 mg loading dose (LD) followed by a 75 mg once-daily MD for 7–9 days, and ticagrelor was administered as a 180 mg LD followed by a 90 mg twice-daily MD for 7–9 days. Subjects received aspirin 75–100 mg once-daily, which was maintained at a constant dose throughout the study. To evaluate the onset of anti-platelet effect, platelet reactivity was assessed at baseline prior to the LD and at 0.5, 2 and 8 h after the LD; to evaluate the effect of the MD, platelet reactivity was assessed just prior to, 2, and 8 h after the last morning dose, and 12 h after the last evening dose of ticagrelor and 24 h after the last morning dose of clopidogrel. Blood samples to analyse the plasma concentrations of ticagrelor and its active metabolite, AR-C124910XX, were drawn at the same time as platelet reactivity assessment.

### Platelet reactivity measurement

Platelet reactivity was assessed using the VerifyNow P2Y12 test, which measures adenosine diphosphate-induced platelet aggregation as an increase in light transmittance and reports values in P2Y12 reaction units (PRU). A higher PRU reflects greater platelet reactivity [4]. Although study treatment was open-label, the PRU results were blinded to study personnel.

### Definitions and endpoints

The primary endpoint was the inhibition of the platelet P2Y_12_ receptor at 2 h after the LD, as measured by least squares (LS) means difference in PRU. Secondary endpoints included the PRU at 0.5 and 8 h after the LD; the PRU at 2, 8 h, and the end-of-dosing interval of the MD (12 h after last evening dose for ticagrelor or 24 h after last dose of clopidogrel); and the percent reduction of PRU from baseline at the time-points measured, i.e. (1 − [PRU after study drug/PRU at baseline]) × 100.

### Statistical analysis

Categorical variables are reported as counts and percentages, and continuous variables as mean ± standard deviation (SD). The primary analysis of the difference in PRU between ticagrelor and clopidogrel at 2 h after the LD was performed using a mixed-effect model with fixed effects for period, treatment sequence, treatment, and a random effect for patient within sequence. Mean on-treatment reactivity was estimated using LS means and two-sided 95 % confidence intervals (CIs). Distribution assumptions underlying the analysis were assessed by residual plots. Secondary analyses of on-treatment reactivity at other timepoints were performed with similar mixed effects models. Several sensitivity analyses were performed. In one pre-specified analysis, platelet reactivity at baseline was included as a fixed effect. In addition, a post hoc analysis was performed including treatment periods in which the baseline on-treatment reactivity prior to study drug administration was <150 PRU, which was thought to be due to incomplete washout of study drug.

A sample size of 12 subjects was required to provide 90 % power to detect a difference in on-treatment reactivity of 100 PRU between ticagrelor and clopidogrel at 2 h post-LD, assuming a SD of 93 PRU, a correlation of 0.5 between paired observations, and a two-sided alpha level of 0.05. Based on a need to enrol a cohort of sufficient size for clinical credibility and to evaluate P2Y_12_ receptor inhibition at secondary timepoints and to collect potential adverse events, it was planned that 34 subjects would be enrolled in order to ensure 28 subjects were evaluable. This would provide more than 99 % power to detect the anticipated primary outcome effect.

## Results

**Fig. 2:**
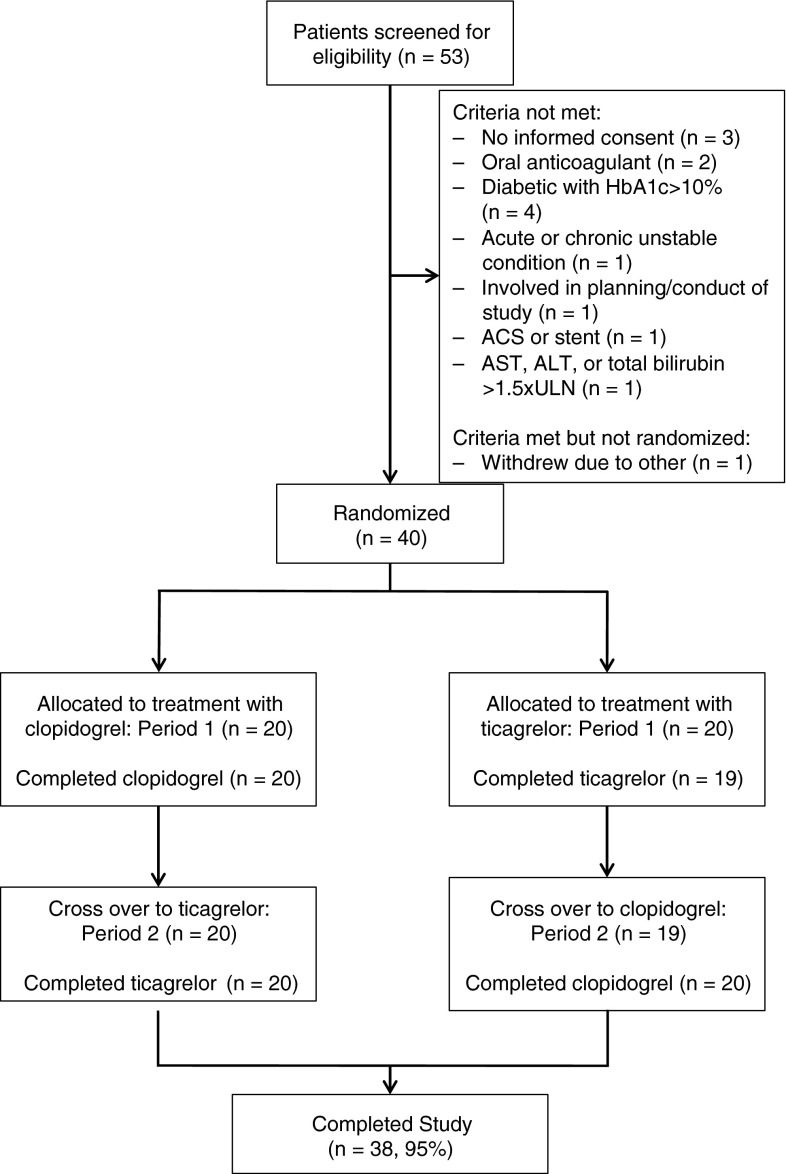
Study flow. A total of 40 subjects were randomly assigned to a treatment sequence, of which 39 completed at least one follow-up visit and of which 38 completed at least 7 days of the maintenance dosing phase for both study drugs

Study flow is shown in Fig. 2. A total of 40 subjects were randomised. All subjects received at least one dose of ticagrelor and 39 subjects received at least one dose of clopidogrel. A total of 38 subjects completed the study. Clinical characteristics and demographics of the randomised subjects are shown in Table 1. The mean age was 63.8 ± 8.8 years, 28 subjects (70 %) were male, 21 (53 %) had diabetes mellitus, and 26 (65 %) had a prior myocardial infarction. Data from three subjects with baseline on-treatment reactivity <150 PRU were excluded from the primary analysis, as this observation was felt to be consistent with an incomplete washout from a P2Y_12_ antagonist and/or the presence of an interfering agent. These values were included in a post hoc sensitivity analysis.

**Table 1:** Demographics and clinical characteristics of the study population

	Total (*N* = 40)
Age (years), mean ± SD	63.8 ± 8.8
Age ≥ 65 (years), *n* (%)	18 (45)
Male sex, *n* (%)	28 (70)
Hypertension, *n* (%)	38 (95)
Dyslipidaemia, *n* (%)	39 (98)
Diabetes mellitus, *n* (%)	21 (53)
Body mass index, mean ± SD	30.2 ± 5.3
Heart failure, *n* (%)	3 (8)
Prior myocardial infarction, *n* (%)	26 (65)
Prior percutaneous coronary intervention, *n* (%)	32 (80)

*SD* standard deviation

### Pharmacodynamic effects of ticagrelor and clopidogrel

**Fig. 3:**
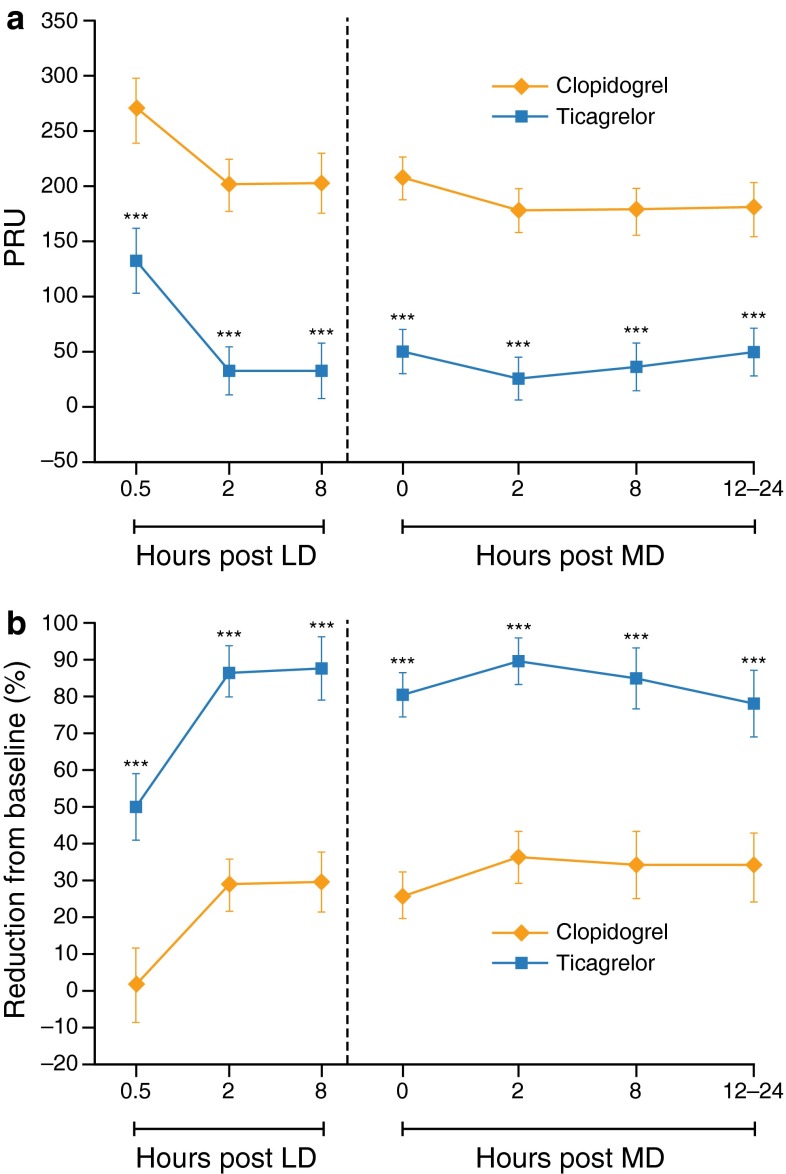
On-treatment platelet reactivity on ticagrelor and clopidogrel in Hispanic subjects with CAD receiving low-dose aspirin. **a** On-treatment reactivity at baseline and after a ticagrelor 180 mg LD or clopidogrel 600 mg LD and after 7–9 days of MD therapy with ticagrelor 90 mg twice daily or clopidogrel 75 mg once daily. **b** Percentage reduction from baseline in on-treatment reactivity after a ticagrelor 180 mg LD or clopidogrel 600 mg LD and after 7–9 days of MD with ticagrelor 90 mg twice daily or clopidogrel 75 mg once daily. *Values* are expressed as the least square means and 95 % confidence intervals. *LD* loading dose, *MD* maintenance dose. ***P < 0.001

The antiplatelet effect of study drug LD is shown in Table 2 and Fig. 3. At 2 h post-LD, the primary endpoint of the study, on-treatment reactivity was significantly lower after ticagrelor compared with clopidogrel (LS means difference, −167 PRU [95 % CI −197, −137], *P* < 0.001). This greater anti-platelet effect was evident within 30 min after the LD and persisted at 8 h after the LD (Table 2). The antiplatelet effects of ticagrelor 90 mg twice-daily MD and clopidogrel 75 mg once-daily MD after 7–9 days of dosing are shown in Table 3 and Fig. 3. On-treatment reactivity was significantly lower with ticagrelor compared with clopidogrel 2 h and 8 h after the MD, and was significantly lower at the end of the dosing interval (12 h after the last ticagrelor MD and 24 h after the last clopidogrel MD). On sensitivity analysis, the primary results were similar when data from the three subjects with abnormally low baseline reactivity were included (LS means difference at 2 h post-LD between ticagrelor and clopidogrel, –154.4 PRU [95 % CI −187.4, −121.4], *P* < 0.001). The results of other sensitivity analyses were also similar to the primary analysis (see Online Appendix.)

**Table 2:** Comparative antiplatelet effect of ticagrelor 180 mg LD compared with clopidogrel 600 mg LD in Hispanic subjects with CAD

Timepoint	Ticagrelor 180 mg LD	Clopidogrel 600 mg LD	LS means difference	*P* value
Primary endpoint				
2 h post-LD (95 % CI)	34 (12, 56)	201 (179, 224)	−167 (−197, −137)	<0.001
Secondary endpoints				
0.5 h post-LD (95 % CI)	135 (105, 164)	270 (239, 301)	−135 (−172, −98.0)	<0.001
8 h post-LD (95 % CI)	34 (9, 59)	203 (177, 229)	−169 (−204, −134)	<0.001

All measurements are in PRU

*CAD* coronary artery disease, *CI* confidence interval, *LD* loading dose, *LS* least squares, *PRU* P2Y12 reaction unit

**Table 3:** Comparative antiplatelet effect of ticagrelor compared with clopidogrel maintenance dosing in Hispanic subjects with CAD

Timepoint	Ticagrelor 180 mg LD	Clopidogrel 600 mg LD	LS means difference	*P* value
End of dosing interval^a^ (95 % CI)	52 (30, 73)	182 (160, 205)	−131 (−158, −103)	<0.001
2 h after dose (95 % CI)	29 (8, 49)	179 (158, 200)	−151 (−177, −124)	<0.001
8 h after dose (95 % CI)	39 (17, 60)	179 (157, 201)	−140 (−168, −112)	<0.001

All measurements are in PRU. Platelet reactivity was assessed at least 7 days of maintenance dosing (ticagrelor 90 mg twice daily and clopidogrel 75 mg once daily)

*CAD* coronary artery disease, *LD* loading dose, *LS* least squares

^a^12 h after last evening dose of ticagrelor and 24 h after the last morning dose of clopidogrel

### Pharmacokinetic profiles of ticagrelor and its metabolite, AR-C124910XX

The pharmacokinetic (PK) profiles of ticagrelor and AR-C124910XX during the LD and MD phases are shown in Table 4. There was a rapid onset of circulating ticagrelor, and the concentration was greatest at the 2-hour post-LD measurement. After repeated dosing during the maintenance phase of treatment, the mean ticagrelor plasma concentration 2 h after a MD was generally consistent with that at 2 h after the LD.

**Table 4:** Plasma concentrations of ticagrelor and its active metabolite, AR-C124910XX

	Post-LD			Post-MD		
	0.5 h	2 h	8 h	2 h	8 h	End of MD dosing interval
Ticagrelor						
Geometric mean—SD	23.6	126.9	234.7	142.9	151.3	64.6
Geometric mean + SD	1023.2	3976.8	775.2	1975.3	684.5	863.4
Arithmetic mean ± SD	381.8 ± 78.0	1,147.4 ± 644.1	502.1 ± 301.5	747.9 ± 428.7	404.8 ± 289.3	370.5 ± 353.4
AR-C124910XX						
Geometric mean—SD	2.2	39.5	42.8	31.7	36.7	19.9
Geometric mean + SD	58.0	662.9	258.5	554.4	298.4	346.9
Arithmetic mean ± SD	31.8 ± 50.0	243.4 ± 141.5	127.4 ± 57.7	206.9 ± 133.6	135.3 ± 66.6	126.9 ± 71.6

*N* = 37, except 2 h post-LD and the end of MD dosing interval, where *N* = 36. Units are ng/ml. Refer to the methods for a description of how the geometric mean was calculated

End of MD dosing interval was 12 h the last evening dose after 7 days of study drug administration

*LD* loading dose, *MD* maintenance dose, *SD* standard deviation

### Adverse events

There were no serious adverse events, bleeding events, or other adverse events that led to discontinuation of study medication.

## Discussion

This is the first randomised, pharmacodynamic (PD) and PK study to specifically compare the antiplatelet effect of ticagrelor and clopidogrel in a pre-defined and statistically powered population of Hispanic patients with stable CAD. We demonstrate that among Hispanic subjects with stable CAD on low-dose aspirin, a ticagrelor 180 mg LD has a more rapid onset of effect compared with clopidogrel 600 mg LD, and that within 30 min, ticagrelor reduced platelet reactivity to a significantly greater extent than clopidogrel, an effect that persisted during the maintenance phase of treatment. The PK of ticagrelor and its metabolite AR-C124910XX were consistent with these findings.

The antiplatelet onset of ticagrelor and its effect during the maintenance phase compared with that of clopidogrel was previously evaluated by Gurbel et al. [5], who reported no subjects of Hispanic ethnicity. In that study, on-treatment reactivity measured by PRU was significantly lower in the ticagrelor group at all assessed times in the first 24 h after loading and during the maintenance phase. In the current study, the mean on-treatment reactivity with ticagrelor 180 mg LD/90 mg twice-daily MD was significantly lower than clopidogrel 600 mg LD/75 mg once-daily MD at all measured times starting 30 min after the loading dose. Specifically, the mean on-treatment reactivity was 135 PRU lower 30 min after the LD, 167 PRU lower 2 h after the loading dose (*P* < 0.001), and 131 PRU lower at the time of the next scheduled maintenance dose; these differences are similar to the VerifyNow P2Y12 test results reported by Gurbel et al. [5]. Plasma concentrations of ticagrelor and AR-C124910XX were also consistent with those observed in previous studies of ticagrelor. Therefore, although we did not directly compare the PD and PK of ticagrelor in Hispanics with non-Hispanics, the antiplatelet effect of ticagrelor among Hispanics appears to be consistent with that observed among the non-Hispanics that make up the bulk of subjects upon which the prior reported experience is based.

Racial and ethnic disparities in cardiovascular care are important public health issues. For example, Hispanic patients have longer delays to reperfusion than non-Hispanic Whites [6]; non-Whites, including Hispanics, presenting with ACS have worse prognosis [7]; and Mexican-Americans are at higher risk of cardiovascular mortality at younger ages than non-Hispanic Whites [8]. The disparity in cardiovascular care also extends to the conduct of randomised, clinical trials. In the PLATO study there was a trend towards higher overall event rates among 1,237 patients with ACS included in Central/South America, as compared to patients included in Asia/Australia, Europe/Middle East/Africa or North America. However, the overall results for primary efficacy and safety were consistent in patients included in Central/South America [3]. The present study, which demonstrates a fast and consistent effect on platelet aggregation, support the observation from PLATO. PD studies such as the current one provide important confirmatory data that the response to ticagrelor does not appear to differ among this ethnic subgroup. The consistent observations with our PK analysis, which track the PD profile, support these findings. Robust demographic data collection in clinical practice combined with comparative effectiveness studies are required to fully explore whether there are substantial differences in clinical efficacy among population subsets. Further studies are also needed to assess the PD effect and clinical efficacy of antiplatelet agents within ethnicities that have a substantial burden of CAD but are not well-represented in large, randomised, clinical trials.

This study has several limitations. The present study was not designed to examine the relation of clinical outcomes and platelet function. Subjects were self-identified as Hispanic. This follows the policy of the United States Food and Drug Administration for the collection of race and ethnicity, as well as that of the United States Department of Health and Human Services. We evaluated patients with stable CAD, and the PD and PK of ticagrelor and clopidogrel may differ among patients with ACS. Of note, the subjects in this study were a higher-risk CAD cohort, as two-thirds had a prior history of myocardial infarction, 80 % had prior percutaneous coronary intervention, 33 % had prior coronary artery bypass grafting, and diabetes mellitus was present in more than half. The Prevention of Cardiovascular Events in Patients With Prior Heart Attack Using Ticagrelor Compared to Placebo on a Background of Aspirin (PEGASUS)-TIMI 54 trial (clinicaltrials.gov identifier, NCT01225562) will examine the safety and efficacy of ticagrelor in combination with low-dose aspirin in patients with a prior myocardial infarction and an additional risk factor, including diabetes mellitus or multi-vessel disease; therefore, the findings of the current study may provide insight into the anticipated PD and PK of the ticagrelor 90 mg twice-daily MD being examined within that trial.

## Conclusion

Among Hispanic subjects with stable CAD, a ticagrelor 180 mg LD followed by 90 mg twice-daily MD provides a more rapid onset of platelet inhibition and a significantly greater antiplatelet effect compared with clopidogrel 600 mg LD followed by 75 mg once-daily MD during both the loading and maintenance phases of treatment. The PK profiles of ticagrelor and its metabolite AR-C124910XX were consistent with these findings.

## Electronic supplementary material

Below is the link to the electronic supplementary material.

Supplementary material 1 (DOCX 23 kb)
