# Supplementary material for: A randomised trial of the pharmacodynamic and pharmacokinetic effects of ticagrelor compared with clopidogrel in Hispanic patients with stable coronary artery disease
Source: J Thromb Thrombolysis. 2014 Oct 11;39(1):8–14. doi: 10.1007/s11239-014-1135-9 (PMC4300410; doi:10.1007/s11239-014-1135-9)

Online appendix

Clinical sites:

Scripps Green Hospital, La Jolla, CA;

Saint Luke's Lipid and Diabetes Research Center, Kansas City, MO;

IRC, Towson, MD; Research Physicians Network Alliance, Hollywood, FL;

South Florida Research Group, Miami, FL; New Jersey Heart, Linden, NJ;

University of Southern California, Los Angeles, CA;

University of Florida, Jacksonville, FL.

Inclusion criteria

For inclusion in the study, patients had to fulfil all of the following criteria:

1. Provision of informed consent prior to any study specific procedures.

2. Female and/or male aged 18 years or older.

3. Documented stable CAD fulfilling any of the following, and taking 75 mg to 100 mg ASA daily treatment:

- Stable angina pectoris (current or history of) with objective evidence of CAD.

- Previous myocardial infarction (MI) history.

- Previous revascularization history, (i.e. PCI or coronary artery bypass grafting).

4. Females were required to be post-menopausal or surgically sterile.

5. Post-menopausal women – women over 50 years of age were considered post-menopausal if they had been amenorrhoeic for 12 months without an alternative medical cause following cessation of all exogenous hormonal treatment.

6. Women must have had a negative urine pregnancy test.

7. Self-identified as Hispanic.

8. For inclusion in the genetic research for exploratory analyses, patients must have signed the informed consent for genetic research. The patient was to be excluded from this genetic research if a previous bone marrow transplant had been performed.

Exclusion criteria

Any of the following was regarded as a criteria for exclusion from the study:

1. Any indication for oral anticoagulant (e.g., atrial fibrillation, mitral stenosis, or prosthetic heart valve) or dual antiplatelet treatment (e.g., clopidogrel, prasugrel, ASA dose other than 75 to 100 mg daily) during the study period.

2. Concomitant therapy with strong CYP3A inhibitors, CYP3A substrates with narrow therapeutic index, or strong CYP3A inducers within 14 days and during study treatment:

- Strong inhibitors: ketoconazole, itraconazole, voriconazole, telithromycin, clarithromycin, nefazadone, ritonavir, saquinavir, nelfinavir, indinavir, atanazavir.

- Substrates with narrow therapeutic index: cyclosporine, quinidine.

- Strong inducers: rifampin/rifampicin, phenytoin, carbamazepine.

3. Increased bleeding risk including:

- Recent (within 30 days) GI bleeding.

- Any history of intracranial, intraocular, retroperitoneal, or spinal bleeding.

- Recent (within 30 days of dosing) major trauma.

- Sustained uncontrolled hypertension (systolic blood pressure [SBP] >180 mmHg or diastolic blood pressure [DBP] >100 mmHg).

- History of hemorrhagic disorders that could have increased the risk of bleeding, e.g. haemophilia, von Willebrand’s disease.

- Inability to discontinue required concomitant therapy with non-steroidal anti-inflammatory drug (NSAID) at screening.

- Patients who had used within 30 days of screening, any oral or parenteral antithrombotic agent.

- Platelet count less than 100,000 mm^3^ or haemoglobin <10 g/dL.

4. Diabetic patients with HbA1c ≥10 %.

5. Contraindication or other reason that clopidogrel, ASA, or ticagrelor could not be administered (e.g. hypersensitivity, active bleeding, major surgery within 30 days of dosing, any bleeding tendency [coagulation defects], acute or chronic liver disease etc.).

6. History of drug addiction or alcohol abuse in the previous 2 years.

7. Patient required dialysis.

8. Participation in another investigational drug or device study within 30 days of dosing.

9. Recent (within 30 days of dosing) blood donation.

10. Patients that were scheduled for revascularization (e.g. PCI, CABG) during the study period.

11. Any acute or chronic unstable condition in the past 30 days or other condition which, in the opinion of the investigator, may have either put the patient at risk or influenced the result of the study (e.g. active cancer, risk for non-compliance, risk for being lost to follow-up).

12. History of moderate or severe hepatic impairment.

13. Patients who in the opinion of the investigator would have been at risk for bradycardia.

14. Involvement in the planning and conduct of the study (applies to AstraZeneca or delegate staff, and study site staff).

15. Previous enrollment or randomization of treatment in the present study.

16. Current smokers, including the use of tobacco containing products in the past 1 month.

17. Patients who had ACS or stent placed within 12 months of screening.

18. A suspected/manifest infection according to the World Health Organization (WHO) risk categories 2, 3, and 4.

19. Aspartate aminotransferase (AST), alanine aminotransferase (ALT) or total bilirubin >1.5 x upper limit of the reference range.

20. Currently taking ticlopidine and cilostazol.

21. History of intolerance or allergy to ASA, clopidogrel, prasugrel, ticagrelor.

Sensitivity analyses

| Platelet reactivity at 2-hours after ticagrelor and clopidogrel LD  *Excluding two patients with missing baseline PRU, and adds PRU at baseline as a fixed effect.* | |
| --- | --- |
| PRU | 2 hours post loading dose |
| LS mean (95 % CI) |  |
| Ticagrelor | 35.3 (15.0, 55.6) |
| Clopidogrel | 197.1 (175.9, 218.3) |
| Difference in LS Means (95 % CI) | -161.8 (-191.2, -132.4) |
| *P*-Value | <0.001 |

*PRU* P2Y12 reaction unit; *LD* loading dose; *LS* least squared

| Platelet reactivity at 2-hours after ticagrelor and clopidogrel LD  *Includes all data from three patients with low baseline reactivity (PRU<150)* | | |
| --- | --- | --- |
| PRU | 2 hours post loading dose | |
| LS mean (95 % CI) |  | |
| Ticagrelor | 34.3 (10.3, 58.3) | |
| Clopidogrel | 188.7 (164.7, 212.7) | |
| Difference in LS Means (95 % CI) | -154.4 (-187.4, -121.4) | |
| *P*-Value | <0.001 | |

*PRU* P2Y12 reaction unit; *LD* loading dose; *LS* least squared


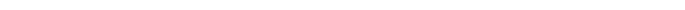

Supplement: Supplementary file 1 — Supplementary material 1 (DOCX 23 kb) [file 11239_2014_1135_MOESM1_ESM.docx]
